# Myrcene and terpene regulation of TRPV1

**DOI:** 10.1080/19336950.2019.1654347

**Published:** 2019-08-26

**Authors:** C. Jansen, L.M.N Shimoda, J.K. Kawakami, L. Ang, A.J. Bacani, J.D. Baker, C. Badowski, M. Speck, A.J. Stokes, A.L. Small-Howard, H Turner

**Affiliations:** a Laboratory of Immunology and Signal Transduction, Chaminade University, Honolulu, HI, USA; b Department of Chemistry, Chaminade University, Honolulu, HI, USA; c Undergraduate Program in Biology, Chaminade University, Honolulu, HI, USA; d Department of Biology, Chaminade University, Honolulu, HI, USA; e Laboratory of Experimental Medicine, John A. Burns School of Medicine, Honolulu, HI, USA; f GBSciences, Las Vegas, NV, USA

**Keywords:** Myrcene, terpenes, TRPV1, calcium entry, pain

## Abstract

Nociceptive Transient Receptor Potential channels such as TRPV1 are targets for treating pain. Both antagonism and agonism of TRP channels can promote analgesia, through inactivation and chronic desensitization. Since plant-derived mixtures of cannabinoids and the *Cannabis* component myrcene have been suggested as pain therapeutics, we screened terpenes found in *Cannabis* for activity at TRPV1. We used inducible expression of TRPV1 to examine TRPV1-dependency of terpene-induced calcium flux responses. Terpenes contribute differentially to calcium fluxes via TRPV1 induced by *Cannabis*-mimetic cannabinoid/terpenoid mixtures. Myrcene dominates the TRPV1-mediated calcium responses seen with terpenoid mixtures. Myrcene-induced calcium influx is inhibited by the TRPV1 inhibitor capsazepine and Myrcene elicits TRPV1 currents in the whole-cell patch-clamp configuration. TRPV1 currents are highly sensitive to internal calcium. When Myrcene currents are evoked, they are distinct from capsaicin responses on the basis of I_max_ and their lack of shift to a pore-dilated state. Myrcene pre-application and residency at TRPV1 appears to negatively impact subsequent responses to TRPV1 ligands such as Cannabidiol, indicating allosteric modulation and possible competition by Myrcene. Molecular docking studies suggest a non-covalent interaction site for Myrcene in TRPV1 and identifies key residues that form partially overlapping Myrcene and Cannabidiol binding sites. We identify several non-*Cannabis* plant-derived sources of Myrcene and other compounds targeting nociceptive TRPs using a data mining approach focused on analgesics suggested by non-Western Traditional Medical Systems. These data establish TRPV1 as a target of Myrcene and suggest the therapeutic potential of analgesic formulations containing Myrcene.

## Introduction

Components of plant secondary metabolomes have been used in traditional and indigenous medical systems for pain management for centuries. Examples include Chinese Traditional Medical System formulations, Japanese Kampo, African systems, indigenous Pacific and Oceanic systems and Indian Ayurveda [1–3]. Numerous countries have pluralized medical systems where these therapies are integrated to varying degrees with Western medical approaches[4], and they form significant components of medical care in economically disadvantaged countries by necessity [3,5]. The evidence base for efficacy of these complex mixtures of medicinal compounds ranges from historical and traditional knowledge, contemporary anecdotal and patient-reported outcomes (PRO), and some (often limited) testing using Western paradigms of randomized controlled trials and mechanistic studies [6,7]. The general relegation of these pharmacopeias to the nutraceutical market in the West has led to the dominance of the debate around “medical” marijuana as the primary plant-based medicine currently under scrutiny in the US and other Western countries for its efficacy and safety [8–11]. Pain is one of the most common indications for the use of *Cannabis* medicinally[12], and there is data supporting its efficacy and its potential as an opioid-sparing approach [13–18].

The use of whole plant *C. sativa* extracts obtained from dispensaries as “medicine” is beset by issues of psychoactive adverse effects (due to the presence of Δ9-tetrahydrocannabinol, THC), lack of consistency and standardization, contamination (microbial, pesticide), and inadequate evidence of efficacy [19–24]. All of these place patients at risk and limit the potential utility of *Cannabis*-derived compounds as therapeutics. There are unmet needs to evaluate the major components of the *Cannabis* secondary metabolome (several hundred cannabinoids and terpenes), discriminate active therapeutic from inactive or dispensable compounds, and reformulate single compounds or mixtures for prescription using accepted regulatory pathways[25]. Both cannabinoid and terpene components of *Cannabis* mixtures need to be included in these analyses since bioactive molecules with therapeutic potential are found in both of these categories [26,27]. In a parallel paper, we assess the cannabinoid suite and in this study, we focused on terpenes. These compounds are often referred to as a part of the “entourage”, a concept where minor components of the *Cannabis* secondary metabolome are proposed to contribute additively or synergistically to physiological effects of the more abundant cannabinoids such as THC, cannabinol and cannabidiol [28–30].

In this study, we focus specifically upon one target for analgesia, the Transient Receptor Potential ion channel TRPV1. Several members of the TRP superfamily of non-selective cation channels have been identified both as nociceptive receptors for pungent plant compounds (e.g., capsaicin, allicin, menthol) and as targets for cannabinoids [31–40]. Both antagonism and agonism of the TRP channel are critical pharmacological approaches for pain management. For example, TRPV1 antagonism has utility in acute analgesia. However, chronic pain management requires longer-term strategies such as receptor and neuronal desensitization using TRPV1 agonists. There is a need to explore whether other naturally-occurring TRPV ligands could cause desensitization at the tissue or cellular level without the accompaniment of high pain levels (such as those caused by the topical 8% capsaicin treatment that is standard in the field) in the short term. The separation of excitatory and analgesic effects of chronic TRPV1 activation is a goal in this field.

In the current paper, we used primary data from a third-party testing laboratory to arrive at a list of 10 terpenes that are of high relative abundance in commonly used medicinal *Cannabis* chemovars [41,42]. We tested each compound for its ability to induce calcium influx into a heterologous system with isolated expression of TRPV1. A mixture of these compounds in strain-equivalent ratios activated large TRPV1-dependent calcium responses. Most of the compounds were inactive when tested singly, with only Myrcene and Nerolidol causing TRPV1 activation. Most of the mixture effect was accounted for by Myrcene, and so we studied this compound in more detail using whole-cell patch-clamping. Myrcene is an activator of a highly rectifying conductance, which is dependent upon the presence of TRPV1 protein. Myrcene-induced currents are distinct from those induced by capsaicin in the magnitude of the attained I_max_ and the fact that Myrcene currents do not transition to the pore-dilated highly permeant state characterized by a linear I/V relationship that has been described for TRPV1. Myrcene-induced currents are highly sensitive to internal calcium levels, and appear rapidly inactivating in a manner dependent on the degree of cytosolic buffering of calcium. Under varying conditions of internal calcium myrcene can be a productive (induction of nA currents and obvious calcium influx) or non-productive (receptor occupancy but minimal current development) ligand for TRPV1, which may open interesting possibilities for therapeutic manipulation of TRPV1 using this compound. Myrcene pre-application and residency at TRPV1 appears to negatively impact subsequent responses to TRPV1 ligands such as Cannabidiol, indicating allosteric modulation and possible competition by Myrcene. Molecular docking studies suggest a non-covalent interaction site for Myrcene in TRPV1 and identifies key residues that form partially overlapping Myrcene and Cannabidiol binding sites. A key terpene moiety that is predictive of likely interaction with the Myrcene site are identified, providing a means to differentiate between compounds within the broad range of *Cannabis* terpenes that are likely to mimic Myrcene.

## Materials and methods

### Cell culture

HEK TRexTRPV1 (human or rat) were cultured in DMEM, 10% Fetal Bovine Serum, 2 mM L-glutamine, 10 μg/ml Blasticidin (Calbiochem, San Diego CA), 400 μg/ml Zeocin (InvivoGen, San Diego CA), transgene expression was induced using 1 μg/ml Tetracycline for 16–24 h. Human TRPV1 and rat TRPV1 were compared for terpene responses and quantitative (e.g., response size) but not qualitative differences in Ca^2+^ response profiles were observed. rTRPV1 was used in the experiments shown here. A comparison of conservation of all key residues in h and r TRPV1 is shown in Table II.

### Chemicals, reagents, and stimulations

General chemicals were from VWR (West Chester, PA) and Sigma Aldrich (St. Louis, MO). Terpenes, beta-Myrcene, Capsaicin, and Capsazepine were from Sigma Aldrich.

### Mixture design

***(1) Terpene profiling.*** Terpenoid analyses (DigiPath Laboratories) were carried out on an Agilent 7890B GC/7697A Headspace/5977A mass spectrophotometer with a DB-624UI and Agilent 5181–8818 split/splitless liner. Injector port temperature was 250°C with a transfer line, valve oven and needle temperature of 180°C. Carrier gas was helium at a flow of 33.0 cm/s. The MS detector was set to scan with a range from 50 to 300 m/z. The instrument was controlled by Agilent Masshunter quantitative analysis (Vers. B.08.00 Build 8.0.593.0). Certified reference standards were from Restek (Bellefonte, PA) with Masshunter library confirmation.

***(2) Strain-inspired mixture***: Cannabidivarin (CBDV), Cannabichromene (CBC), Cannabidiol (CBD), Cannabidiolic Acid (CBDA), Cannabigerol (CBG), Cannabigerolic Acid (CBGA), Cannabinol (CBN), alpha-Bisabolol, alpha-Humulene, alpha-Pinene, beta-Caryophyllene, beta-Myrcene, (+)-beta-Pinene, Camphene, Limonene, Linalool, Nerolidol. ***(3) Cannabinoid mixture***: Cannabidivarin (CBDV), Cannabichromene (CBC), Cannabidiol (CBD), Cannabidiolic Acid (CBDA), Cannabigerol (CBG), Cannabigerolic Acid (CBGA), Cannabinol (CBN). ***(4) Terpene mixture***: alpha-Bisabolol, alpha-Humulene, alpha-Pinene, beta-Caryophyllene, beta-Myrcene, (+)-beta-Pinene, Camphene, Limonene, Linalool, Nerolidol.

### Calcium assay (bulk method)

Cells were washed and incubated with 0.2 μM Fluo-4 [43] for 30 min at 37°C in a standard modified Ringer’s solution of the following composition (in mM): NaCl 145, KCl 2.8, CsCl 10, CaCl_2_ 10, MgCl_2_ 2, glucose 10, Hepes·NaOH 10, pH 7.4, 330 mOsm. Cells were transferred to 96-well plates at 100,000 cells/well and stimulated as indicated. Calcium signals were acquired using a Flexstation 3 (Molecular Devices, Sunnydale, USA). Data were analyzed using SoftMax® Pro 5 (Molecular Devices). Where indicated, nominally calcium-free external conditions were achieved by the preparation of 0 mM CaCl_2_ Ringer solution containing 1mM EGTA.

### Electrophysiology

Cells grown on glass coverslips were transferred to the recording chamber and kept in a standard modified Ringer’s solution of the following composition (in mM): NaCl 140, KCl 2.8, CaCl_2_ 1, MgCl_2_ 2, glucose 10, Hepes·NaOH 10, pH 7.2, with osmolarity typically ranging from 295 to 325 mOsm. Intracellular pipette-filling solutions contained (in mM): Cs-glutamate 140, NaCl 8, MgCl_2_ 1, Cs-BAPTA 10, HEPES·CsOH 10, pH 7.2 adjusted with CsOH with an osmolarity ranging from 295 to 325 mOsm. Patch-clamp experiments were performed in the tight-seal whole-cell configuration at 21–25°C. High-resolution current recordings were acquired by a computer-based patch-clamp amplifier system (EPC-9, HEKA, Lambrecht, Germany). Patch pipettes had resistances between 2 and 4 MΩ after filling with the standard intracellular solution. Immediately following establishment of the whole-cell configuration, voltage ramps of 50 ms duration spanning the voltage range of – 100 to +100 mV were delivered from a holding potential of 0 mV at a rate of 0.5 Hz over a period of 500 s. All voltages were corrected for a liquid junction potential of 10 mV between external and internal solutions because of glutamate use as intracellular anion. Currents were filtered at 2.9 kHz and digitized at 100 µs intervals. Capacitive currents and series resistance were determined and corrected before each voltage ramp using the automatic capacitance compensation of the EPC-9. The current development graphs were generated by extracting currents at −80 and +80 mV. Where applicable, statistical errors of averaged data are given as means ± SEM with n determinations.

### Molecular modeling

Software used was Molecular Operating Environment 2018 by Chemical Consulting Group. The TRPV1 structural source file was the rat crystal structure RCSB PDB No. 5IS0 (EMD-8119), electron microscopy structure (at 3.43 Angstrom)[44]. The protein with ligand deleted was protonated and minimized with Amber10 constrained with rigid water molecules. Site Finder settings were: Probe Radius 1 = 1.4, Probe Radius 2 = 1.8, Isolated Donor/Acceptor 3, Connection Distance 2.5, Minimum Site Size 3, Radius = 2, Rendered Alpha Spheres. Docking at site 4 (Ala 400 through ASP 707) used dummy atoms at alpha spheres. The placement method was Alpha PMI (Principal Moment of Inertia), scored with ASE (Alpha Sphere and Excluded volume-based ligand-protein docking) and refinement using Induced Fit as the method and scored with ASE.

### Analysis

Results are shown as the mean ± standard deviation. Statistical significance was determined based on Student’s t-test or ANOVA. Adjacent to data points in the respective graphs, significant differences were recorded as follows: single asterisk, p < 0.05; double asterisk, p < 0.01; triple asterisk, p < 0.001; no symbol, p > 0.05. Experiments are all *n* of at least 3.

## Results

### Strain-inspired and cannabinoid or terpene mixtures initiate calcium responses in a TRPV1 over-expression system

**Figure 1.**
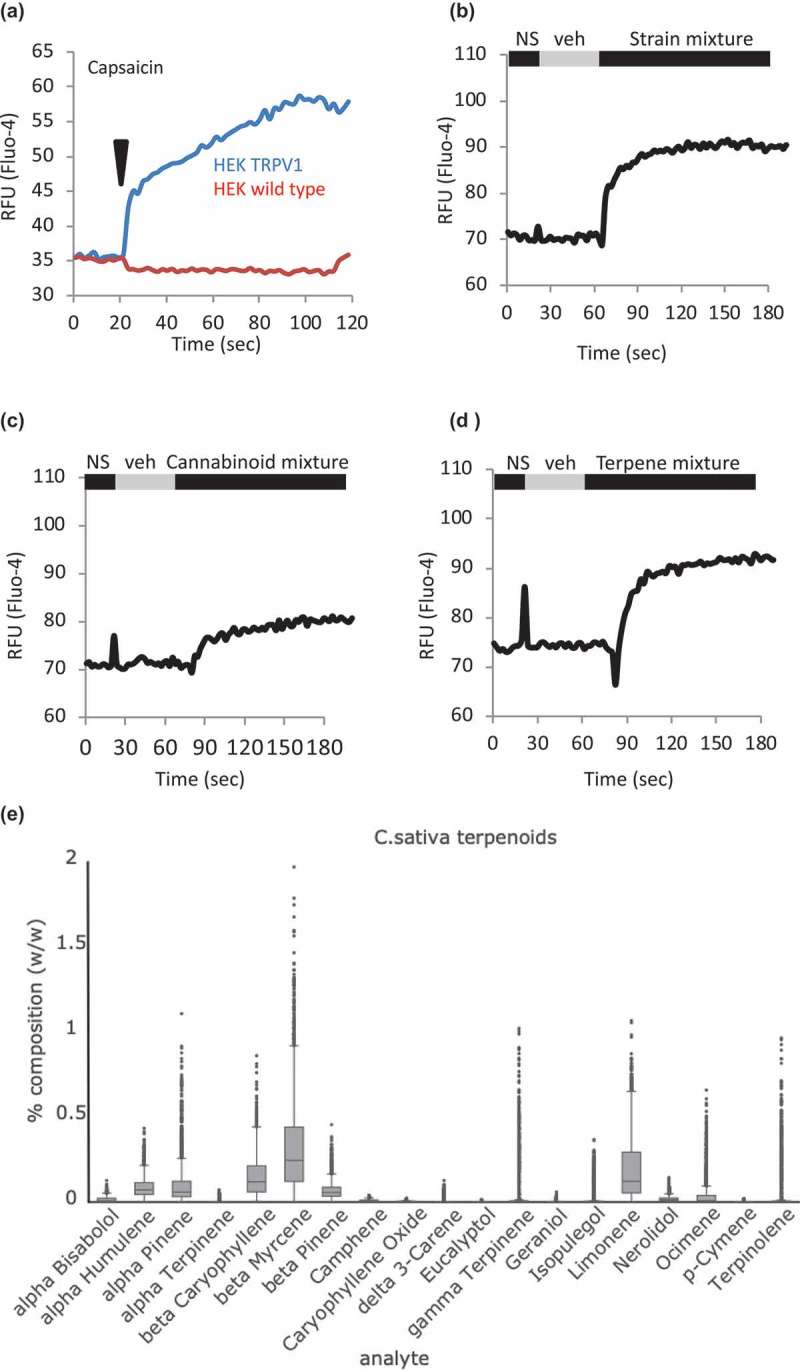
**Strain-inspired and cannabinoid or terpene mixtures initiate calcium responses in a TRPV1 over-expression system**. a. **HEK-TRPV1 differentiate TRPV1-dependent calcium responses**. HEK WT and HEK-TRPV1 were loaded with Fluo-4 and population-based calcium assays were conducted in the presence of 1mM external calcium. After a non-stimulated (NS) period to establish a baseline, cells were stimulated at 20 s with 100 nM capsaicin. b, c, d. HEK-TRPV1 were exposed to a matched vehicle mixture (veh) or the indicated mixtures of cannabinoids plus terpenes, cannabinoids, or terpenes, as indicated. Fluo-4 calcium assays were performed as described. e. **Distribution of content values of terpenoids in 2,662 *Cannabis* samples representing 396 “strains” in three major chemovars** [41,42]. The box plots display the range and distribution of each analyte in terms of %w/w. The line bisecting each box represents the median for that distribution. The lower and upper lines show the minimum and maximum values of the lower and upper quartiles, respectfully. The points show the outliers for the sampled ranges. The y-axis values represent % (w/w) of dried flower for each of the indicated cannabinoid species.

We evaluated *Cannabis* constituents for their ability to mobilize intracellular free calcium in HEK cells that were either untransfected (wild type, WT) or bearing tetracycline-inducible expression of TRPV1 (HEK-TRPV1). We first confirmed (Figure 1(a)) that this system resolved TRPV1-dependent calcium fluxes, which were present in HEK-TRPV1 but not HEK WT when stimulated using the TRPV1 ligand capsaicin[45]. We built a complex mixture of cannabinoids and terpenes which, omitting tetrahydrocannabinol in order to focus on non-psychoactive components, recapitulated the composition of an example strain of medical marijuana in current use in Nevada (DigiPath Laboratories) [41,42]. This “strain mixture” was assembled in two ways, either using 10 micromolar as a final dose of each constituent or setting the cannabinol (the second major constituent after THC) at a nominal dose of 10 micromolar and adding relative amounts of other constituents at ratios informed by quantitative data on strain composition obtained by a third-party testing laboratory (see Figure 1(e)). In both cases, extensive optimization of the mixtures in terms of appropriate vehicles for each component was performed, and vehicle controls exactly matched the diluents present in the applied mixtures without active compounds added. No major differences were seen between the presumably saturating 10 micromolar doses (Figure 1(b)) and the ratioed doses (not shown) and so the former were selected for subsequent experiments. Figure 1(b) shows the calcium-mobilizing effect in HEK-TRPV1 of mixtures containing seven cannabinoids (Cannabidivarin (CBDV), Cannabichromene (CBC), Cannabidiol (CBD), Cannabidiolic Acid (CBDA), Cannabigerol (CBG), Cannabigerolic Acid (CBGA), Cannabinol (CBN)), and 10 terpenes (alpha-Bisabolol, alpha-Humulene, alpha-Pinene, beta-Caryophyllene, beta-Myrcene, (+)-beta-Pinene, Camphene, Limonene, Linalool, Nerolidol). Figure 1(c,d) shows the effects in HEK-TRPV1 of the 7 cannabinoids and 10 terpenes as separate mixtures. These are population-based Ca^2+^ assays where each datapoint represents the mean of triplicate samples of 100,000 cells per point. Figure 1(e) shows, for reference, the relative abundancies (% w/v) of each terpene we considered for inclusion in our study based on 352 separate chemotype analyses of chemovars currently in patient use [41,42]. Some compounds were excluded on the basis of solubility or stability issues, resulting in the list of 10 terpenes evaluated here.

### Individual terpenes contribute differentially to terpene mixture-induced calcium responses

In a separate study, we evaluated individual effects of cannabinoids. Cannabidiol, Cannabividarin, Cannabigerolic Acid, Cannabichromene, and Cannabidiolic Acid were found to be effective TRPV1 ligands both in bulk calcium assay and using electrophysiology[46]. In this study, we focused on terpenes, and Figure 2(a–l) show calcium responses to individual terpenes applied to HEK-TRPV1, with a summary in Figure 2(m). These are population-based Ca^2+^ assays where each datapoint represents the mean of triplicate samples of 100,000 cells per point. Responses are present in cells stimulated with Myrcene and Nerolidol, suggesting that these compounds may be primarily driving the large calcium mobilization responses seen in response to the terpene mixture. Responses to Nerolidol are significantly smaller than those to Myrcene, and Figure 2(n,o) show that Myrcene explains most of the response to the terpene mixture in HEK-TRPV1 cells.

**Figure 2.**
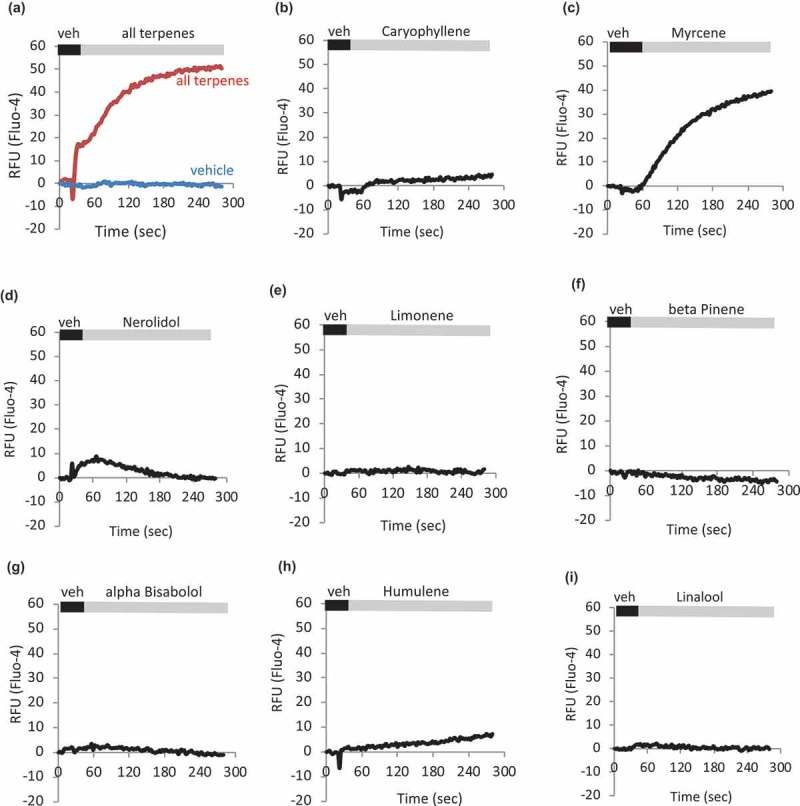
**Individual terpenes contribute differentially to terpene mixture-induced calcium responses**. a-l. HEK-TRPV1 were loaded with Fluo-4 and population-based calcium assays were conducted in the presence of 1mM external calcium. After a matched vehicle exposure (veh) period to establish a baseline, cells were stimulated at 20 s with the indicated terpenes (a. all terpenes, 10 μM; b-l. indicated terpene at 10 μM). m. **Summary of** a-l. Area under the curve analyses were performed followed by Student’s t-test. P-value relative to vehicle: Caryophyllene, Limonene, Bisabolol, Linalool, Humulene, Pinene, Camphene, Ocimene; p > 0.05. Myrcene, p = 0.00055. Nerolidol, p = 0.002. n. **Relative contribution of Myrcene compared to response to mixture of all Terpenes**. o. **Area Under the Curve (AUC) analysis comparing Myrcene alone, Terpene mixture, and Terpene mixture without Myrcene (all compounds at 10 μM final) to matched vehicles**. HEK-TRPV1 were loaded with Fluo-4 in the presence of 1mM external calcium. Calcium flux data were collected for 900 s and AUC calculated.

**Figure 2.**
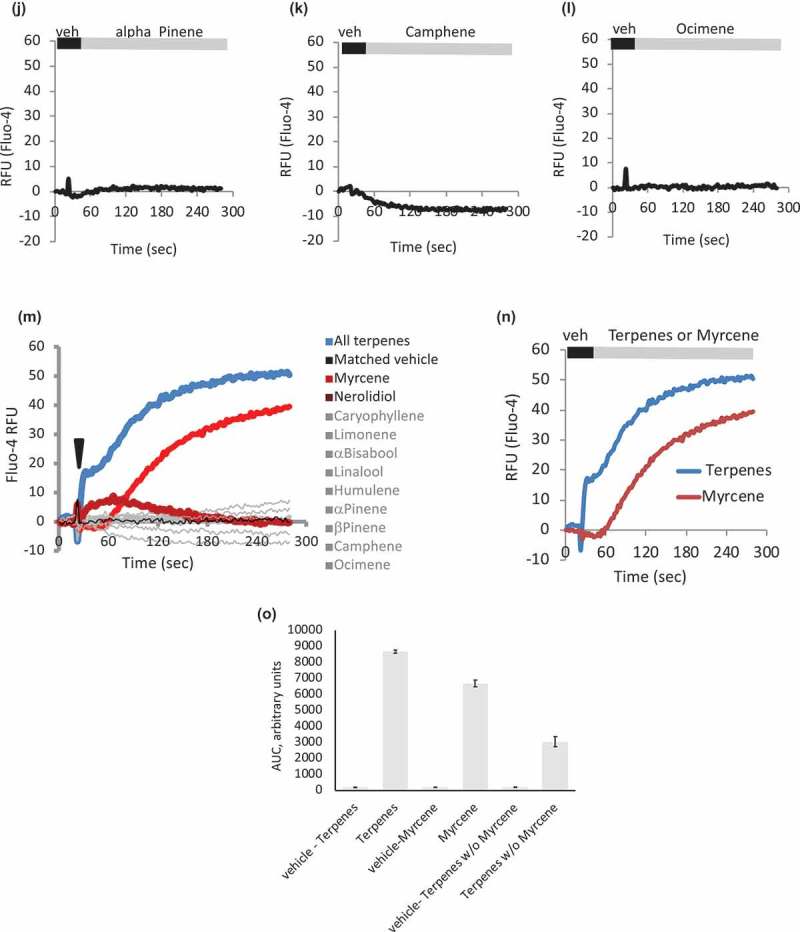
(Continued)

### Dependency of terpene and myrcene responses on presence of TRPV1

**Figure 3.**
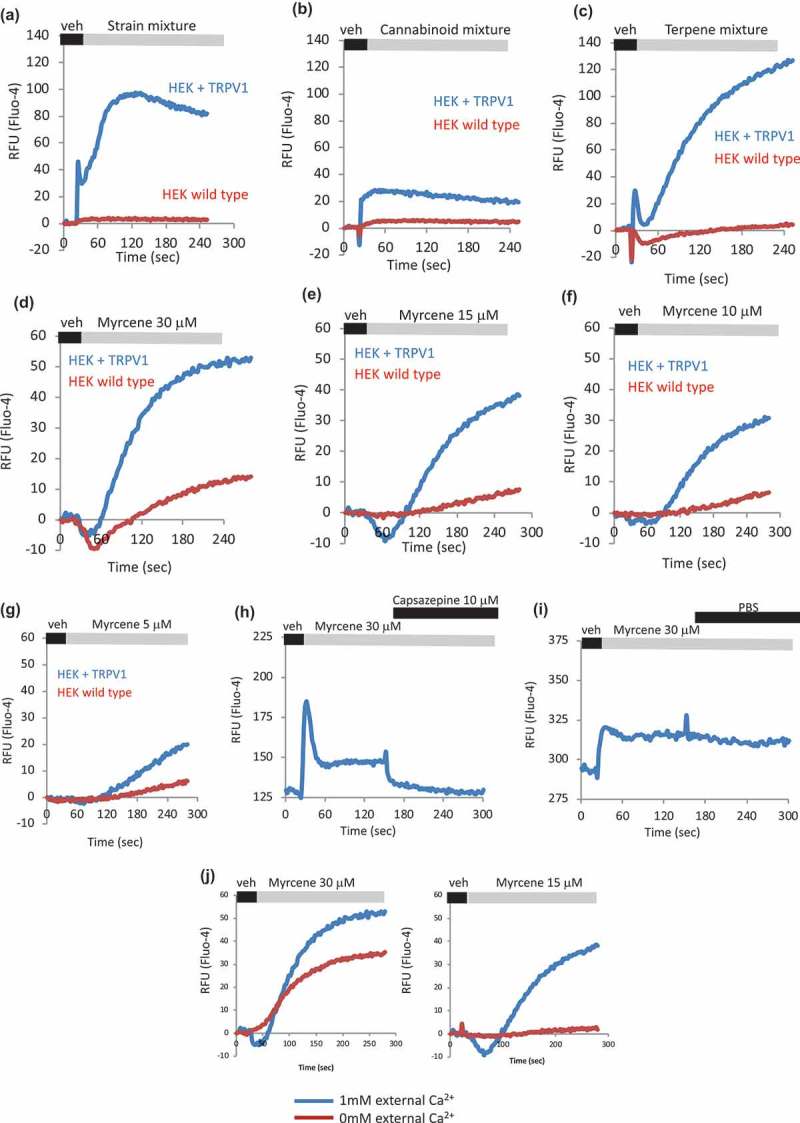
**Dependency of terpene and myrcene responses on presence of TRPV1**. a–c. HEK WT and HEK-TRPV1 were loaded with Fluo-4 and population-based calcium assays were conducted in the presence of 1mM external calcium. After a non-stimulated (NS) period to establish a baseline, cells were stimulated at 20 s with the indicated compound or mixture. d-g. Myrcene dose-response. Area under the curve analyses were performed followed by Student’s t-test. P value for HEK TRPV1 relative to HEK WT: panels A–G, P < 0.005. h, i. Effect of TRPV1 antagonist Capsazepine on calcium responses initiated by Myrcene in HEK-TRPV1 cells. Area under the curve analyses were performed followed by Student’s t-test. P value for trace in panel H relative to I: P = 0.002. j. Comparison of Myrcene induced (two doses, left and right panels) calcium responses in HEK TRPV1 stimulated in the absence (red trace, 0 mM added CaCl_2_ plus 1 mM EGTA) or presence of external calcium. Area under the curve analyses were performed followed by Student’s t-test. P-value for 1mM relative to 0mM external Ca^2+^: left panel, P = 0.018; right panel, P = 0.0008.

We used the comparison between HEK WT and HEK TRPV1 cells to establish which, if any, of the calcium responses observed were dependent on the presence of TRPV1. Figure 3(a–c) show that the strain-inspired, cannabinoid and terpene mixtures described above are without significant calcium-mobilizing effects in HEK WT, but when cells are induced to express TRPV1, calcium-mobilizing responses are observed. Figure 3(d–g) shows a dose-response of Myrcene in HEK WT and TRPV-expressing cells, and again, the observed calcium mobilization responses are mainly dependent on the presence of TRPV1. The low-level responses seen in Figure 3(d), for example, may represent another endogenous TRP involvement (possibly TRPV2, data not shown). The relationship between Myrcene and TRPV1 was further confirmed in Figure 3(h,i) where the TRPV1 antagonist capsazepine was applied after Myrcene addition, causing a rapid drop-off in the calcium response (top panel). In contrast, when the capsazepine vehicle (PBS) was added in the same protocol (bottom panel) no diminution in the Myrcene-induced calcium response was seen. Finally, we asked whether all of the calcium mobilization observed in HEK-TRPV1 treated with Myrcene represented entry across the plasma membrane or whether there was a store release component. TRPV1 present in internal membranes (e.g., ER and Golgi during biosynthesis and trafficking) can gate calcium stores as it is being trafficked to the cell surface and if the applied ligand is sufficiently lipophilic to access internal membranes[47]. Figure 3(j) shows that under nominally calcium-free external conditions (0 mM added CaCl_2_ plus 1mM EGTA), Myrcene does cause stored calcium release. Interestingly (Figure 3(j) right panel *cf* Figure 3(e)), the release response appears less dose-sensitive than the composite release and influx response, perhaps reflecting that a component of specific activity for Myrcene at internal TRPV1 is access across membranes and the cytosol.

### Myrcene activation of cationic currents is dependent on TRPV1

**Figure 4.**
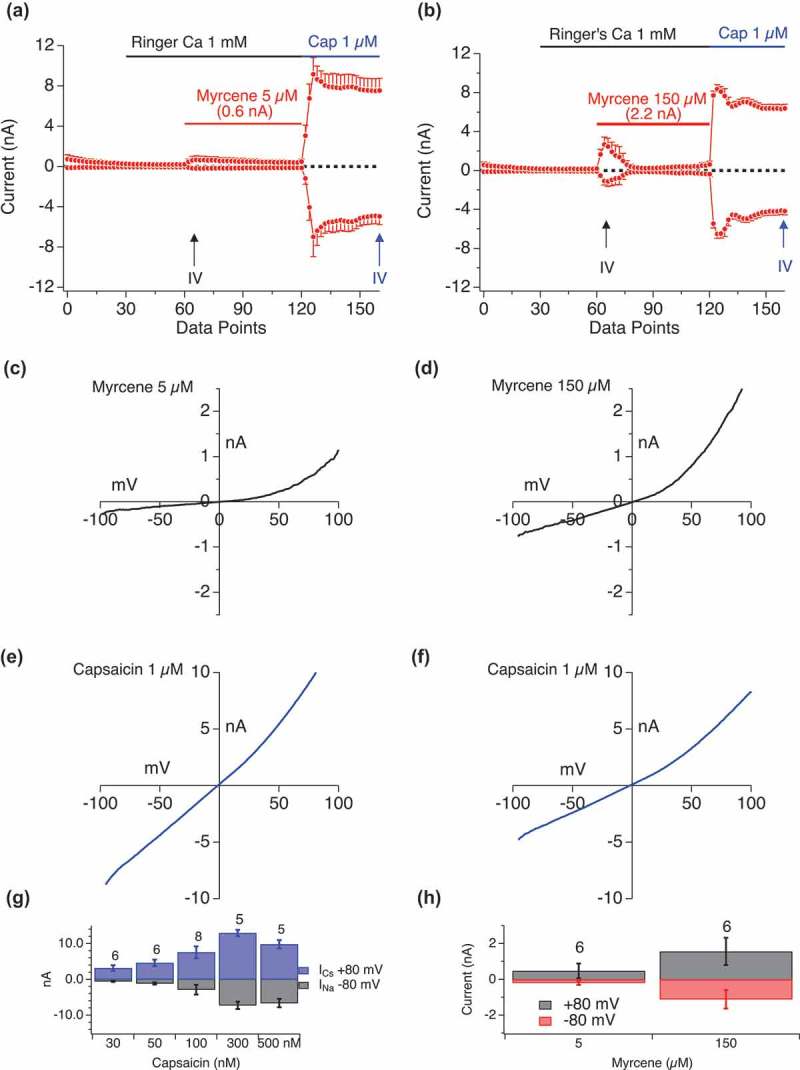
**Myrcene activation of cationic currents is dependent on TRPV1**. a, b. Current development graphs (n = 6) for HEKTRPV1 stimulated as indicated with Myrcene at 5 μM or 150 μM followed by capsaicin (1 μM). Recordings were performed in 1mM external Ca^2+^. Attained I_max_ are shown above the Myrcene recordings. The current development graphs were generated by extracting currents at −80 mV and +80 mV. c–f. **High dose Myrcene responses do not elicit pore-dilation in TRPV1 as assessed by rectification in the I/V relationship**. c, d. Extracted I/V curves for Myrcene responses. Extraction point from the time series is indicated by upward arrows on a and b. e, f. Extracted I/V curves for Capsaicin responses. Extraction point from the time series is indicated by upward arrows on a and b. High amplitude Myrcene-induced currents (4 nA) were induced using 150 μM Myrcene application. These rapidly inactivating currents display a rectification that is not seen in large capsaicin-induced TRPV1 currents where pore dilation has occurred. g, h. Bar graphs representing inward and outward current at different doses of Capsaicin (G) and Myrcene (H).

Myrcene is a component of Thyme oil, some components of which are TRPV1 and TRPA1 activators (thymol, linalool) [48,49]. However, the target of Myrcene as an ion channel activator [50–52] has not been explicitly explored. We used whole-cell patch-clamping to investigate Myrcene activation of TRPV1. Figure 4(a,b) shows current development over time in HEK-TRPV1 cells exposed extracellularly to the indicated doses of Myrcene using an applicator pipette in the whole-cell patch-clamp configuration. Current-voltage relationships were extracted and (Figure 4(c,d)) are consistent with published characteristics of TRPV1 prior to state transition (see below). Capsaicin-induced currents are shown for comparison (Figure 4(e,f)).

### State-specific activation of TRPV1 by myrcene

TRPV1 is a two state channel, exhibiting rapid pore dilation after activation in response to Capsaicin [53–55]. This pore-dilation results in a loss of selectivity, rendering the channel permeant to large cations (e.g., NMDG) and in this state, the I/V relationship becomes highly linearized and reverses at close to 0 mV potentials. Currents at the largest Imax that we see with Myrcene (e.g. Figure 4(c,d)) retain their rectifying characteristics. In contrast, capsaicin-induced currents immediately exhibit the dilated state (Figure 4(e,f)). Note that the Myrcene-induced currents shown here in response to a moderate ligand dose (five micromolar, selected to minimize calcium-induced current inactivation, see below), are nevertheless of the order of 1–1.2 nA. They are relatively dwarfed by the massive TRPV1 currents present in this over-expression system, but are sizable in their own right. Figure 4(g,h) presents the rectification properties of Myrcene-induced TRPV1 and Capsaicin-induced TRPV1 both before and after state transition. We note that lower Myrcene doses induce a slow inactivating current while at higher concentrations the current is fast inactivating, thus suggesting either different modes of action or that the Ca^2+^ entry resulting from higher activation translates to more rapid Ca^2+^ -dependent inactivation. The latter is not explored further in this paper but we examine Ca^2+^ -dependent inactivation below.

### External calcium impact on myrcene activation of TRPV1

**Figure 5.**
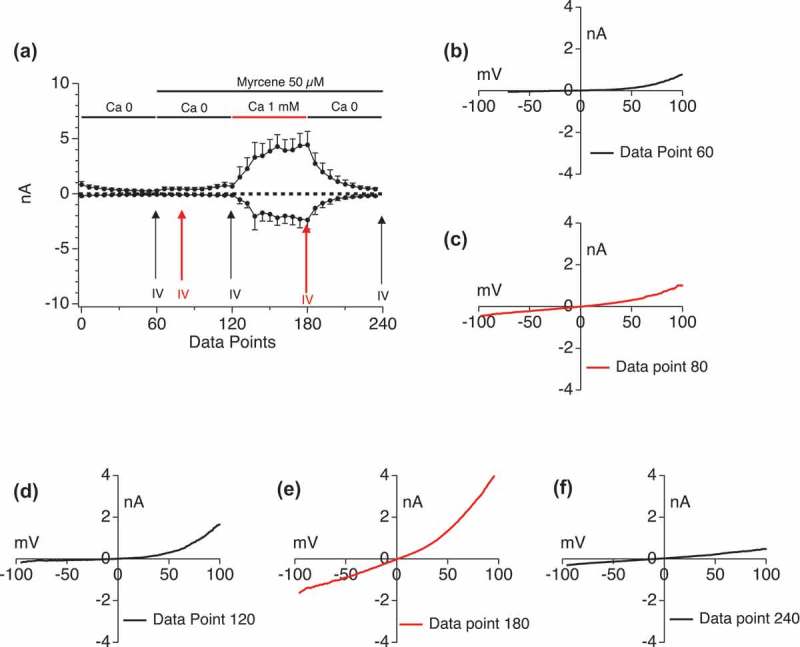
**External calcium impact on Myrcene activation of TRPV1**. External calcium levels in the perfusion buffer were manipulated sequentially in the absence and presence of simultaneously applied Myrcene. a. Current development graphs (n = 6). b-f. Extracted current/voltage relationships at the indicated data points.

In Figure 5 we evaluated the impact of external calcium levels on Myrcene activation of TRPV1. Figure 5(a) shows that a ~ 1 nA conductance develops in the absence of external calcium but when Ca_ext_ is restored to 1 mM, a large 5 nA conductance develops which retains its highly rectifying I/V relationship (extracted I/V curves are shown in Figure 5(b–f)). When external calcium is removed the conductance dissipates rapidly. We note that this experiment also eliminates the possibility that stored Ca^2+^ release (see Figure 3(j)) is activating the Ca^2+^ -release activated current (CRAC) in these cells since the small current activated under zero external Ca^2+^ (60–120 s) in Figure 5(a) does not have the positive reversal potential and rectification properties of I_CRAC_.

### Internal calcium impact on myrcene activation of TRPV1

**Figure 6.**
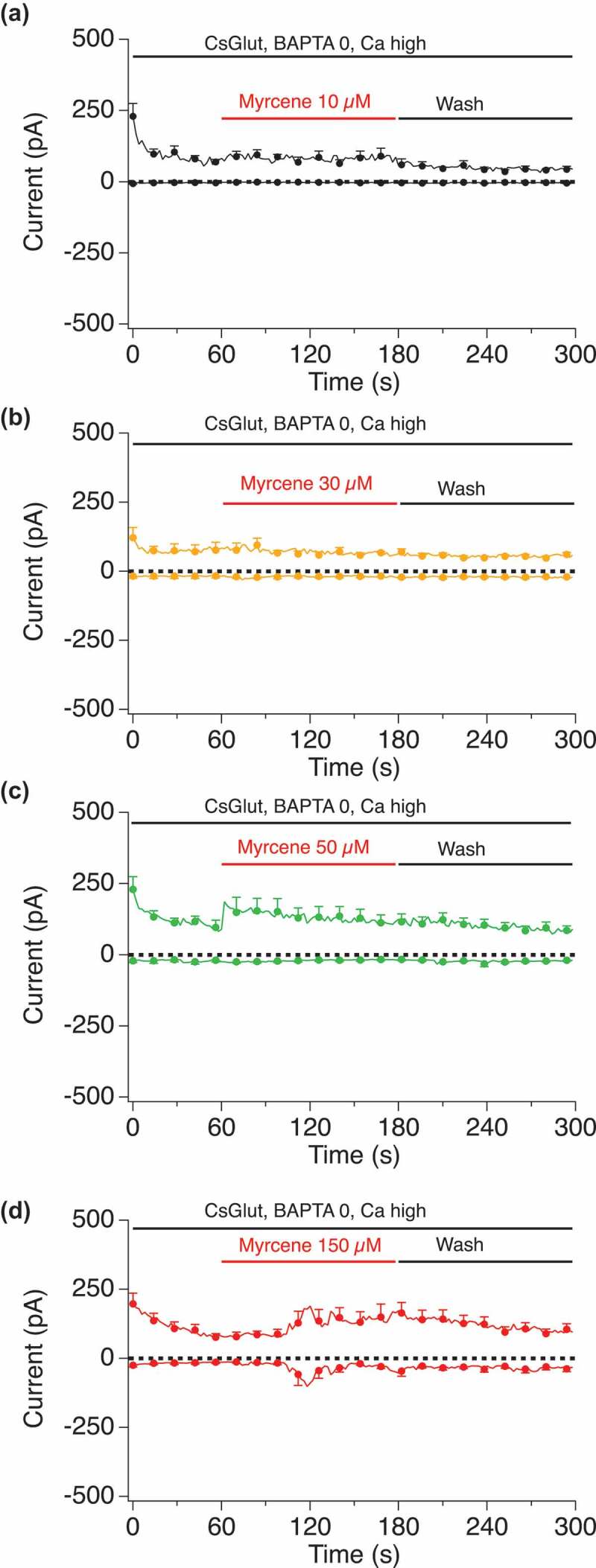
**Dose-response of Myrcene in unbuffered internal calcium conditions**. Current development graphs for the indicated doses of Myrcene recorded with unbuffered calcium in the internal solution. a, n = 4. b, n = 5. c, n = 8. d, n = 8.

**Figure 7.**
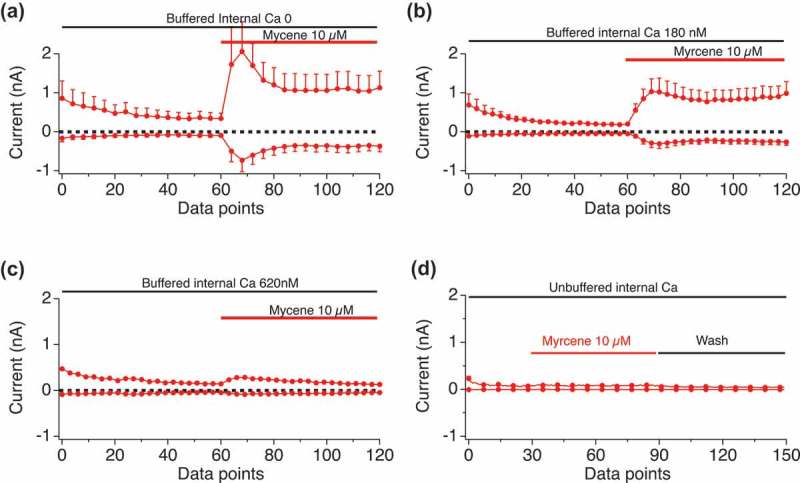
**Internal calcium impact on Myrcene activation of TRPV1**. Current development graphs for the indicated doses of Myrcene recorded in the internal conditions that set cytosolic calcium to the indicated levels (zero, 180 nM, 620 nM, unbuffered). a, n = 6, b, n = 4, c and d, n = 6.

We hypothesized that Myrcene may have a high permeation of external calcium which then desensitizes the channel and prevents activation of the TRPV1 current. To test this, we buffered internal calcium to different levels including Ca 0 nM, Ca 180 nM, Ca 620 nM, and left internal calcium unbuffered with zero mM BAPTA in the internal solution. Figure 6(a–d) shows a dose-response of Myrcene (10 to 150 micromolar) in cells perfused with external solution containing no BAPTA, leaving internal calcium essentially unbuffered. Under these conditions minimal, if any, Myrcene-induced TRPV1 currents were observed. We then altered the internal Ca^2+^ conditions as shown in Figure 7. When internal calcium is buffered to zero (Figure 7(a)) large current manifest in response to Myrcene. Low internal calcium levels that mimic the cytosol at rest (180 nM, Figure 7(b)) allowed Myrcene-activated TRPV1 to manifest at 1–2 nA I_max_, and this effect was diminished as we raised the internal calcium levels to 620 nM (Figure 7(c)). In comparison, Figure 6(d) shows the effect of a very high Myrcene dose (150 μM) in unbuffered cytosolic Ca^2+^ showing that even under such high levels of stimulation the presumed Ca^2+^ dependent inactivation of the channel suppresses currents.

#### Myrcene effects on subsequent TRPV1 ligand application

**Figure 8.**
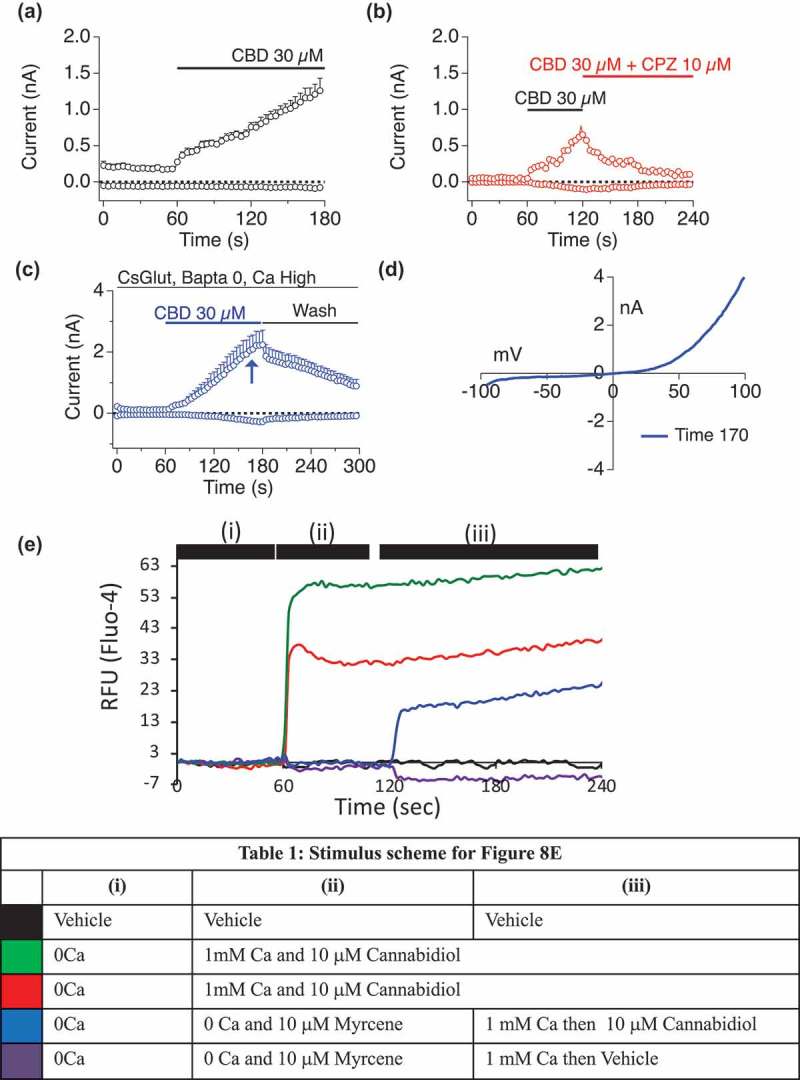
**Myrcene suppression of subsequent TRPV1 ligand responses**.a–d. CBD initiation of TRPV1 conductances in HEK TRPV1.Cannabidiol (CBD), Cannabividarin, Can nabigerolic Acid, Cannabichromene and Cannabidiolic Acid were found to be effective TRPV1 ligands both in bulk Ca^2+^ assay and using electrophysiology [46]. a. CBD induction of outwardly rectifying conductance in HEK TRPV1. b. CBD-induced current is sensitive to application of the TRPV1 inhibitor Capsazepine. c. CBD-induced current is sensitive to washout. d. Current-voltage relationship for CBD-induced TRPV1.e. Fluo-4 Ca^2+^ responses in HEK TRPV1 stimulated according to the schema shown in Table I. All concentrations are in micromolar. For all three protocols, Myrcene was allowed to occupy TRPV1 in the absence of external Ca^2+^ (i). For the second recording phase (ii), 1 mM external Ca^2+^ was restored or Ca^2+^-free conditions were maintained and cannabidiol or myrcene were applied. For the third phase (iii), Ca^2+^ restoration and addition of stimuli were performed for those samples that saw Ca^2+^-free conditions in phase 2.

Myrcene-induced TRPV1 currents are highly sensitive to internal Ca^2+^, raising the interesting possibility that under high internal Ca^2+^ conditions Myrcene could occupy TRPV1, not induce flux, but affect subsequent availability to other stimuli. We have previously shown that Cannabidiol (CBD) is an effective ligand for TRPV1[46], and Figure 8(a–d) exemplify the CBD-mediated effects on TRPV1 including activation of a current that develops to I_max_ of up to 5nA (Figure 8(a)), is sensitive to both capsazepine and washout (Figure 8(b,c)) and is a rectifying current with E_rev_ of ~0mV (Figure 8(d)). We sought to explore the potential for Myrcene application to modulate subsequent CBD effects. Figure 8(e) examines the responses when Myrcene is allowed to occupy the channel in 0 mM external Ca^2+^, prohibiting influx. When a Ca^2+^ “add-back” protocol is performed with Cannabidiol (Figure 8(e), Table I) as a second stimulus in 1 mM external Ca^2+^, the Cannabidiol response is suppressed compared to Cannabidiol without prior Myrcene. These data suggest the Myrcene has the potential to act as an allosteric modulator of other TRPV1 ligands, and prompted an effort to model the interaction sites at TRPV1 for both molecules.

#### Molecular docking of myrcene and cannabidiol at TRPV1

**Figure 9.**
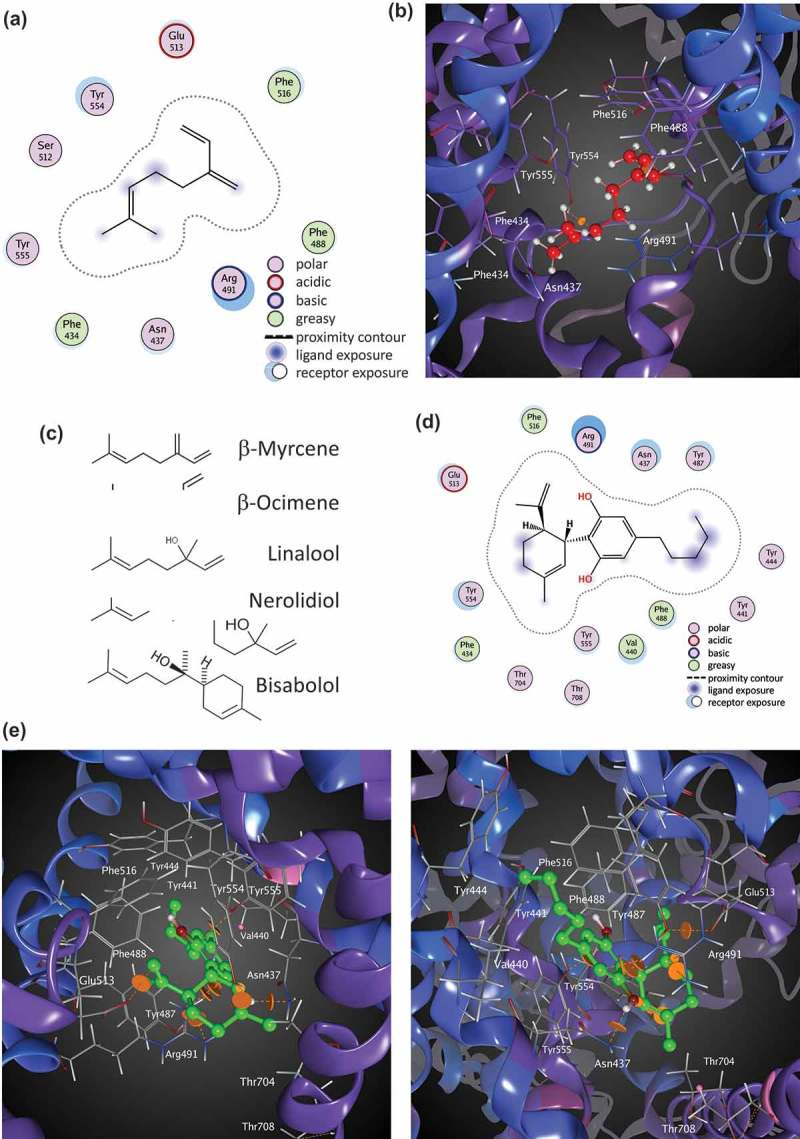
**Molecular docking of Myrcene at TRPV1. A**. Ligand interactions of Myrcene at binding site 4 of TRPV1 (two-dimensional representation). b. Myrcene docked at binding site 4 of TRPV1 (three-dimensional representation). c. Similarities in chemical moieties between specific terpenes found in *Cannabis* and other plant sources. d. Cannabidiol docked at TRPV1 (two-dimensional representation). e. Two views (upper and lower panels) of Cannabidiol binding pocket in TRPV1 (three-dimensional representation).

Small molecule ligands for oxidation-sensitive TRPs such as TRPA1 and V1 can act through electrophilic additions at the same Cysteines that are regulated by hypoxia and hyperoxia [56–58]. However, not all ligands participate in electrophilic additions with the channels. We performed preliminary molecular docking analyses using the Cryo-EM structure of rTRPV1 (RCSB PDB No. 5IS0) to assess potential sites and mechanisms for Myrcene binding. We performed an unbiased computational modeling analysis of Myrcene:TRPV1 compared to Allicin:TRPV1 binding[56] (MOE Site Finder, Molecular Operating Environment version 2018, Chemical Computing Group, Montreal, QC). First, on the basis of its structure, Myrcene is unlikely to participate in the electrophilic additions but is more likely to participate through lipophilic interactions with the channel. Through hydrophobic interactions over 80 potential binding sites for Myrcene were identified in TRPV1, many in the region of Cys 621 but no close interactions with Cys 616 or 621 were observed. One site (site #4) showed binding of both Allicin and Myrcene but a lower (better) docking energy (−17.7 versus −14.0 kcal/mol) for Myrcene over Allicin, despite the fact that Allicin was able to interact with the Cysteines as described, likely in a covalent manner, whereas Myrcene was only able to interact through hydrophobic interactions primarily with Arg 491 and Tyr 554 (Figure 9(a,b)). Other residues implicated in this contact are F488, N437, F434, Y555, S512, E513 and F516 (Figure 9(a,b)). Each of these residues is identical or closely conserved between rat or human TRPV1 and most have been implicated previously as of importance in ligand binding or regulation of TRPV1, for example, prior studies showed a Tyr 554 to alanine mutation ablated both capsaicin and resiniferatoxin binding in TRPV1[59]. These relationships are fully described in Table II and include a protonation site (R491)[45], capsaicin interacting sites and residues that contribute to the hydrophobic interior of transmembranes domains S1,2, and 4 [60]. Several are involved in voltage or thermal sensing [61,62], and based on mutagenesis studies this Myrcene binding site might also be sensitive to Resiniferatoxin competition [62–64]. Residues close to the S4-S5 linker, a key regulatory region for TRPs, are also implicated in binding.

One chemical moiety in Myrcene that is contacted by several residues in the binding site is a dimethyl group that is shared by a number of other terpenes found in *Cannabis* and other plant sources. Figure 9(c) shows a group of *Cannabis* terpenes that share this moiety, and may have the capacity to occupy this site. Interestingly, our data confirm that Nerolidiol also actives TRPV1-mediated Ca^2+^ influx (Figure 2(d)), although we do not see similar fluxes with the other compounds at the doses we tested. Given that other terpenes have dramatically different structures than this group (*cf*. humulene, not shown) our data may offer a pre-screen approach for decisions as to which of the large number of terpene molecules should be prioritized for exploration in the context of TRPV1 and nociception.

**Figure 10.**
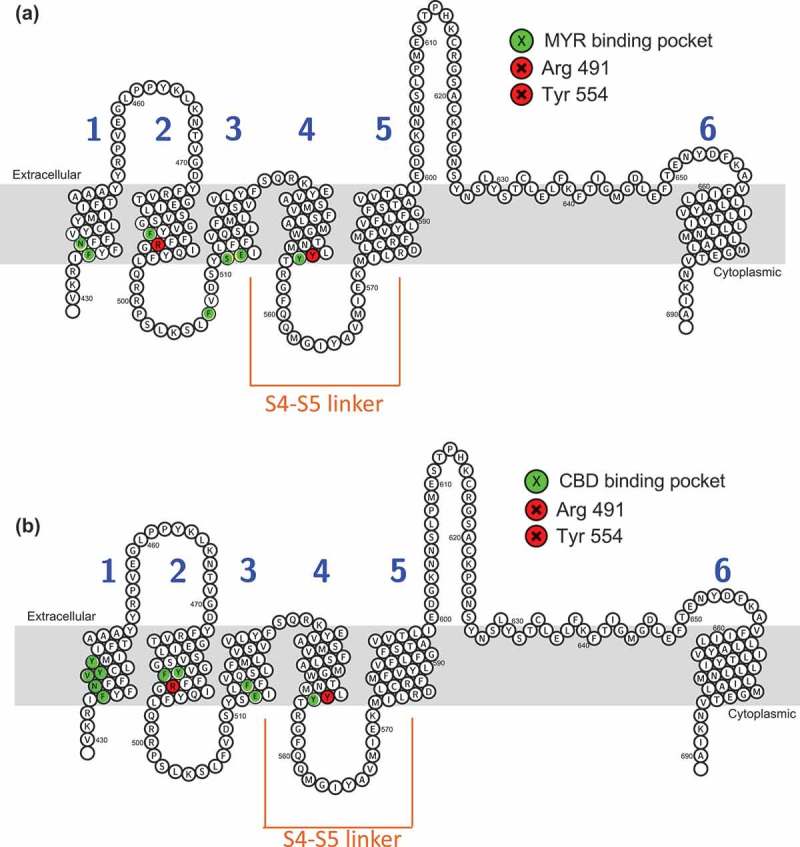
**Residues implicated in Myrcene and Cannabidiol binding to TRPV1 via molecular modeling**. Topology of rTRPV1 with residues implicated in Myrcene (a) and Cannabidiol (b) binding highlighted. Table II. Overview of residues implicated in Myrcene and Cannabidiol binding to TRPV1 using molecular docking relative to prior functional and mutagenesis studies. Non-bonded contacts are coded orange.

We evaluated the CBD binding site similarly (Figure 9(d,e)). A binding pocket partially overlapping with that of Myrcene was identified and the best scoring pose showed a docking score of −26.5 kcal/mol. In this site, Y554 and R491 are important, as for Myrcene. The remaining residues implicated in CBD binding show both similarities and differences to the Myrcene site. Figure 10(a,b) compares the implicated residues in binding Myrcene and CBD across a two-dimensional representation of the channel’s protein sequence. Table II presents each implicated residue, its conservation or identity between rat and human, location to the S4-5 linker, function, effects of mutagenesis where known, and supporting references.

## Discussion

This study evaluated terpenes found in *C. sativa* for their effectiveness in activation of TRPV1. We found that while mixtures of terpenes were effective in eliciting large calcium influxes in a TRPV1-expression system, most of this activity was in fact accounted for by two compounds, Myrcene and Nerolidol. Of these, Myrcene is the most significant contributor to calcium influxes. Myrcene responses showed complete dependence upon the presence of TRPV1 and were blocked effectively by the TRPV1 antagonist Capsazepine. Myrcene currents shared similar properties to those evoked by capsaicin, especially in their dependence on internal and external calcium levels. One distinction, however, was the lack of transition to the pore-dilated, non-rectifying, channel open state that is associated with capsaicin-induced currents [53–55]. Interestingly we also note this lack of pore dilation in TRPV1 responses to CBD, CBN, and CBD [46]. Myrcene has been identified as an analgesic in past studies including those of Traditional Medical System (TMS) approaches. As a component of *Occimum* oils, it has demonstrated anti-neuropathic pain effects in mice, working in combination with eugenol (a TRPA1 ligand)[65]. Analgesic oils from other settings contain Myrcene [66–68,69]. A TCM formulation, the Danggui-Zhiqiao herb-pair, has been indicated for pain and contains Myrcene [70]. In addition, our analyses of two Kampo and two TCM formulations for pain suggest that Myrcene has convergently been arrived at in numerous TMS (Supplemental Table 1) [71–74,75,76,77,78]. In Western studies, Myrcene was shown to be anti-nociceptive in mice [79]. Interestingly in this study, the mechanism of action of Myrcene was thought to be via alpha 2-adrenoreceptors based on antagonism of the Myrcene effect by yohimbine and naloxone. There is a connection between adrenoreceptors and TRPV1, where the adrenoreceptor activation inhibits TRPV1, possibly via activation of a TRPV1-inhibiting PKA-mediated phosphorylation pathway [80]. Further complexity arises when we consider that naloxone inhibition may also indicate opioid receptor involvement, and functional interactions between opioid and TRPV1 receptors have been shown in nociceptive and neuroprotective pathways [58,81–83]. One key aspect for further study is the dose-response relationship for myrcene (and other *Cannabis* components), and TRPV1. The doses used in this study are high due to the solubility issues inherent in working with myrcene. The specific activity and effective dose of myrcene at TRPV1 is likely to be far lower than our experiments suggest, and preliminary studies with carrier molecules indicate that this is the case (ALSH, unpublished).

The complex relationship between intracellular free calcium levels and the degree of Myrcene activation may be of interest when we consider how to leverage Myrcene in analgesic applications. While for many pain applications there is a focus on orally bioavailable TRPV1 antagonists, in applications for indications such as diabetic neuropathy and post-herpetic pain, topical application of high percentage capsaicin creams are a standard approach [84]. These rely on chronic agonism of TRPV1 leading to both desensitization of TRPV1 at the cellular level and the induction of neuronal cell death via large influxes of calcium and sodium through the channel [85,86], but are associated with severely burning sensations. If these cytotoxic fluxes are indeed dependent upon the pore-dilation transition of TRPV1 in response to capsaicin, then Myrcene may not perform as well as Capsaicin in topical applications. Alternatively, if Myrcene may be able to separate excitatory and analgesic effects of TRPV1 application given that it does not inevitably cause state transition of TRPV1, then it may have advantages in terms of side effect profiles. Moreover, Myrcene at certain concentrations may be able to occupy the receptor effectively and cause cellular level desensitization, without appreciable influx. However, it is not yet clear if this is a scenario that is likely to occur physiologically.

The relationship between Myrcene activation of TRPV1 and intracellular calcium levels is also complex. Calcium influx and accumulation in the cytosol rapidly inactivate Myrcene, and when the cytosol is unbuffered (an unphysiological situation that is only found in the patch-clamp) this can effectively prevent manifestation of the current. However, the population-based assays that we show certainly are consistent with large fluxes of calcium entering in response to Myrcene, despite the “free calcium” patch-clamp scenario showing no appreciable current. The basis for these disparate observations may be complex: (1) The comparison between patch-clamp conditions and bulk calcium assays may be “apples to oranges”. Clamping the cytosolic calcium to 180 nM and 620 nM, respectively, represent “resting” and “activated” levels of calcium in the cytosol but do not allow movement around the clamped level. The implications of the measurable but decreased Myrcene-induced TRPV1 at 620nM Ca^2+^_i_ are that the current would still be appreciable during the higher elevation of Ca^2+^_i_ likely during cellular activation. “Free” or unbuffered calcium conditions in the cytosol are not physiological because the cytosol has significant buffering capacity from calcium-binding proteins and calcium sinks [87]. Thus, none of the patch-clamp conditions exactly mirrors those in the population-based assay, but we do know that Myrcene can initiate fluxes and that at certain fixed cytosolic calcium levels that are within the physiological range of increases in Ca^2+^_i_ which occur during cellular activation, Myrcene-induced currents do manifest. (2) We cannot exclude that non-TRPV1 conductances may be contributing to the population-based flux measurements. We assessed TRPM8, V2, and A1 responses to Myrcene, and we see only TRPV2 showing a small Myrcene response (LMNS, HT, unpublished data). We also assessed whether store-operated calcium release via the CRAC (Calcium Release Activated Channel) pathway was occurring, given that Myrcene is gating store release via TRPV1 that is present in the ER or Golgi as part of its biosynthesis and trafficking. HEK wild type cells have small but measurable CRAC. However, we analyzed the small current present in Figure 5(a) under zero calcium external conditions and this current does not have the physiological signature of CRAC, which has a very positive (+60mV) E_rev_ and is highly inwardly rectifying (Figure 5(c)). (3) Since Myrcene does initiate store release it is possible that a significant component of the population-based calcium responses are attributable to release rather than influx. Figure 3(j) supports this, meaning that Myrcene is indeed an effective TRPV1 ligand and in cells with significant intracellular TRPV1 (i.e. in an over-expression context) this leads to intracellular calcium release. The fact that this release is not driving I_CRAC_ is likely related to the fact that specialized high threshold InsP3-sensitive stores and STIM1 coupling are needed for CRAC initiation [88,89]. A remaining open question will be to assess relative importance of store release and influx in an endogenous TRPV1 expression system where there may be less significant accumulation of immature or maturing TRPV1 in biosynthesis and trafficking compartments.

TRPV1 and, and other nociceptive TRPs (A1, V2) are redox sensors [56]. Hyperoxia is sensed by the reduction state of specific Cysteine residues [56]. A commonality of many of the small molecule ligands for these channels that are derived from plant sources is their ability to react with channel Cysteines in electrophilic Michael additions. TRPV1 has a lower range of redox sensitivity [57] than, for example, TRPA1, but some ligands such as allicin affect this channel via covalent modification of the redox-sensing Cysteine residues [90–92]. Similarly, in TRPA1, cysteines form covalent adducts with activating reactive electrophiles (e.g., mustard oil, unsaturated aldehydes, maleimides). However, some ligands for these channels do not work through covalent interaction and some work through covalent interactions with one channel target but not another[48]. Future experiments will need to definitely define whether Myrcene participates in this evolutionarily conserved sensing of electrophilic small molecules [93–95], through investigation of Myrcene effects on TRPV1 proteins that have been mutated to lack key Cysteine targets. However, our preliminary molecular docking data suggest the Myrcene is interacting hydrophobically, non-covalently, and in a manner not strongly dependent on reactive cysteines. Tyr 554 is implicated in the binding site we identify, which has also been implicated in capsaicin binding [59] and is part of the S4-S5 loop between the 4^th^ and 5^th^ transmembrane domains of TRPV1 [61]. The Myrcene binding site we identify will need to be confirmed by mutagenesis but has a number of interesting features. We note also that we cannot exclude that more than one binding site (especially given the variability in current development onset times that we occasionally observe) may exist in TRPV1 for Myrcene. The chemical moiety in Myrcene that is contacted by R491 and Y554 is a group that is shared by a number of other terpenes found in *Cannabis* and other plant sources. Given that other terpenes have dramatically different structures than this group (*cf*. humulene, not shown) the presence/absence of this moiety may help discriminate and prioritize screening for indications such as analgesia that involve TRPV1. The modeling of binding pockets at scale (multiple cannabinoids or terpenes across multiple TRPs) may be of significant utility in categorizing and prioritizing likely activators, desensitizers, inhibitors or allosteric modulators prior to expensive *in vitro* and *in vivo* testing. Since it seems likely that complex mixtures of cannabinoids and terpenes are likely to be of therapeutic importance, understanding which are likely to interact, compete or synergize also be facilitated by this approach. Our data show that Myrcene and CBD share elements of a binding site and can influence one another physiologically, for example.

If Myrcene indeed explains some of the analgesic effects of *C. sativa* preparations, it will be important to determine whether it can substitute for the THC-containing plant in terms of effectiveness and whether it may act additively or synergistically with major or minor, non-THC, cannabinoids in effective mixtures. In particular, it may be of interest to explore cannabinoids/terpenes/other secondary metabolites that affect receptors such as TRPA1 and TRPM8, extending the potential efficacy of a mixture multiple sensory neurons types. Our data suggest that several minor cannabinoids in fact discriminate between TRPV1, TRPA1, and TRPM8 [46]. Given that toxic by-products emerge in some recreational practices that may bleed into the medical marijuana industry, moving these and other products into a regulated supply chain of purified and certified compounds is important [96]. Supply chain issues and reliable sourcing of large quantities of the “entourage” compounds [25] may challenge their application medically if their efficacy becomes proven. For Myrcene at least, engineered production at scale in bacteria may be possible [97]. It will also be important to assess the side effect and adverse effect profile of Myrcene [98–102] and to assign its documented effects in pain and other potential indications (ischemia/reperfusion injury [103], osteoarthritis [104], peptic ulcer [105], anti-oxidant [106]) to TRPV1 or other possible receptors. In this regard, network pharmacological analysis may be of assistance (see Supplemental Table III).
