# Supplementary material for: Myrcene and terpene regulation of TRPV1
Source: Channels (Austin). 2019 Aug 26;13(1):344–66. doi: 10.1080/19336950.2019.1654347 (PMC6768052; doi:10.1080/19336950.2019.1654347)

SUPPLEMENTAL FIGURE 1.

**Table III**	**TMS Pain Formulation**			
	**Kampo**		**TCM**	
	Goshajinkigan	***Goshuyuto***	***Xie Xin Tang***	***Si Mo Tang***
**Plant composition**	Jukujio (*Rehmanniae Radix preparata*), Goshitsu *(Achyranthis bidentatae),* Sanshuyu (Corni fructus), Sanyaku *(Dioscoreae rhizoma),* Shazenshi (*Plantaginis semen.),* Takusha *(Alismatis Rhizoma),*Bukuryo *(Sclerotium Poriae Cocos),* Botanpi *(Paeoniae Moutan Cortex),* Keihi *(Cinnamomi Cortex),* Bushi *(Aconiti Radix lateralis preparata)*	Goshuyu *(Evodiae Fructus),* Ninjin *(Ginseng Radix),* Taiso *(Jujubae Fructus),* Shokyo *(Zingiberis Rhizoma).*	Hange *(Pinelliae Rhizoma),* Ogon *(Scutellariae Radix),* Oren *(Coptidis Rhizoma),* Ninjin *(Ginseng Radix),* Kankyo *(Zingiberis Siccatum Rhizoma),* Kanzo *(Glycyrrhizae Radix),* Taiso *(Jujubae Fructus).*	Lignum Aquilariae Resinatum *(Aquilaria agallocha Roxb)* Arecae Semen (*Areca catechu),* Ginseng *(Panex notoginseng).*
**Bioactive compounds**	374	373	498	208
**Convergent Compounds**	123<br>16<br>28			
**Nocioceptive TRP ligands represented in compound sets:** Myrcene, piperine, camphor, vanillin, cinnamaldehyde, geraniol, eugenol, gingerol, ginsenosides				

SUPPLEMENTAL FIGURE 2.

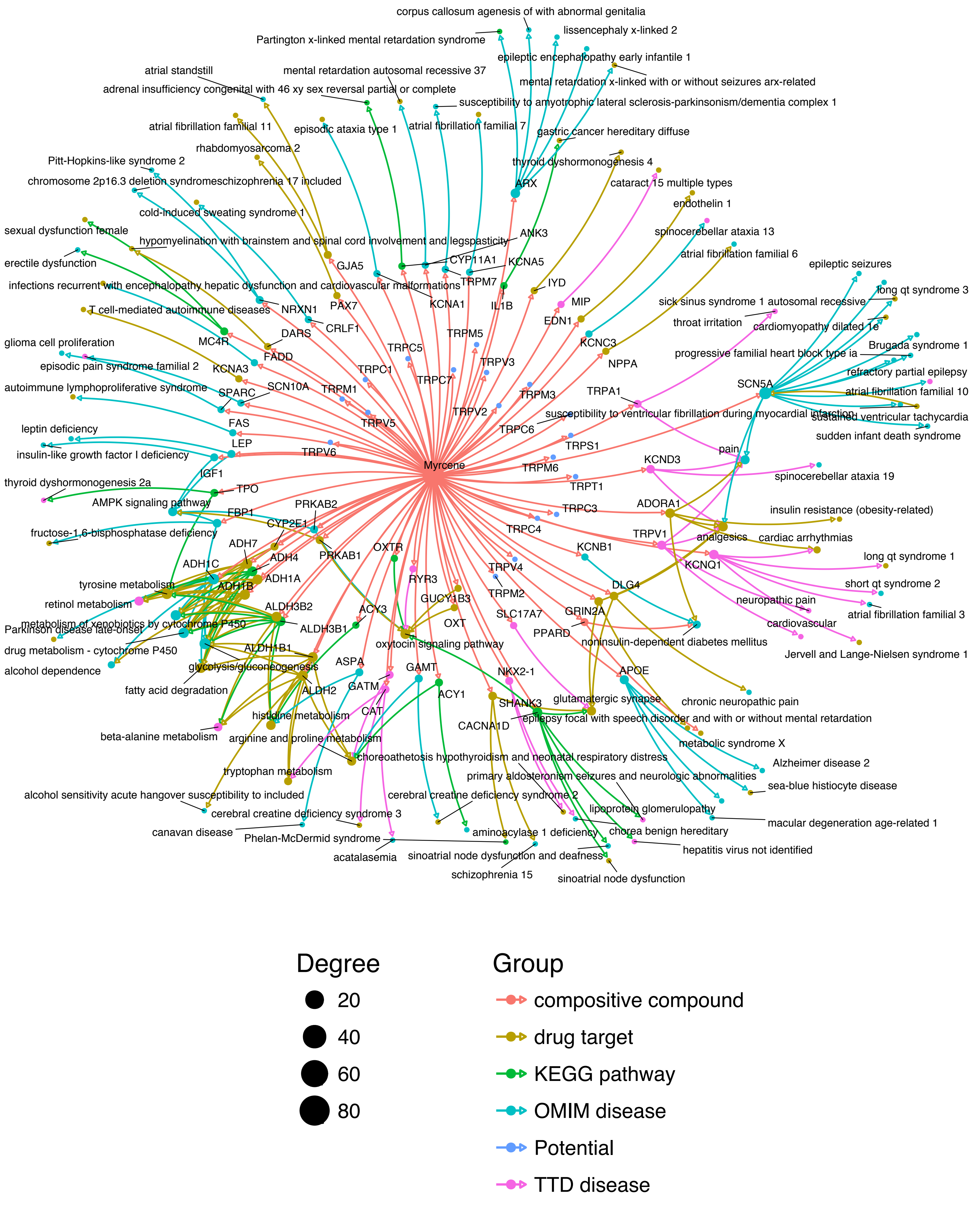

Supplement: Supplemental Material [file kchl-13-01-1654347-s001.pdf]
